# Testing MD-Link, a Low-Cost Mobile Electrocardiography Monitoring Device, in Patients With Irregular Heartbeat: Protocol for a Cross-Sectional Study

**DOI:** 10.2196/resprot.8762

**Published:** 2019-01-31

**Authors:** Precious J Kilimo, Tai Le, Ngoc T Phan, Huy-Dung Han, Hoc T Hoang, Nguyen C Vu, Nga TT Pham, Hung Cao, Cuong K Nguyen

**Affiliations:** 1 Dartmouth College Hanover, NH United States; 2 University of California, Irvine Irvine, CA United States; 3 Institute of Population, Health and Development Hanoi Viet Nam; 4 Hanoi University of Science and Technology Hanoi Viet Nam; 5 Vietnam National Heart Institute Hanoi Viet Nam

**Keywords:** cardiac arrhythmia, cardiovascular diseases, ECG, electrocardiography, irregular heartbeat, mHealth, mobile devices, mobile phone

## Abstract

**Background:**

Having mobile devices that provide patients with the ability to record and monitor the electrical activity of their heart enhances patient self-care and the early detection of irregular heartbeat (cardiac arrhythmia), yet few such devices exist in Vietnam. Challenges exist for introducing mobile electrocardiography (ECG) monitoring devices in Vietnam, including patient accessibility and affordability. A low-cost mobile ECG monitoring device designed and developed in Vietnam, which allows patients to easily measure their heart’s electrical activity and navigate recordings, may be a solution.

**Objective:**

The aim of this project is to assess the usability of the MD-Link system, a newly developed mobile handheld 1-lead ECG device, in detecting patients with irregular heartbeat. We will compare its outputs to the standard printed outputs of a 12-lead electrocardiogram generated by the Nihon Kohden Cardiofax S Electrocardiograph Model ECG-1250K.

**Methods:**

We will conduct a cross-sectional study in two stages, including the measurement of ECG signals of patients using the MD-Link and the Nihon Kohden Cardiofax S and analysis of the selected standard outputs collected from the ECG recordings of the MD-Link and the Nihon Kohden Cardiofax S. The MD-Link consists of (1) a mobile device (eg, a smartphone); (2) a lead wire with 2 disposable electrodes; and (3) an easy-to-use mobile app interface enabling the upload and accurate display of ECG recordings to patients and their clinicians. Our research team, consisting of members from Dartmouth College; the Institute of Health, Population and Development; Hanoi University of Science and Technology; and physicians and nurses from Thanh Chan Clinic, will assist in carrying out this project.

**Results:**

We will proceed with a publication plan that includes a project report and, ultimately, articles for peer-reviewed journals. We also hope to disseminate our work at relevant conferences to provide more coverage and exposure to the MD-Link mobile device. Recruitment and data collection were completed in January 2018. Data analysis started in February 2018 and is ongoing. Results are expected mid-2019.

**Conclusions:**

At the end of this project, we will have developed and tested the MD-Link, a low-cost mobile ECG monitoring device, with some supportive comparisons to standard ECG devices commonly used in heart clinics or hospitals in Vietnam. Our long-term goal is for the MD-Link to be easily accessible, affordable, and to fit into a patient’s daily routine, thus improving the care and treatment of patients with cardiovascular diseases (CVDs).

**International Registered Report Identifier (IRRID):**

RR1-10.2196/8762

## Introduction

### Background

Noncommunicable diseases, particularly cardiovascular diseases (CVDs), are currently the leading cause of death globally, with an estimated 17.7 million people dying annually [1,2]. Combined effects of population growth, aging of populations, and epidemiological changes in CVDs have resulted in increasing global deaths from CVDs [3]. Among these diseases, myocardial infarction (heart attack) and stroke account for 80% of all the CVD deaths [2].

In Vietnam, there has been an epidemiological change in which the overall morbidity and mortality patterns have shifted from communicable to noncommunicable diseases [4,5]. A recent global burden of disease study shows that cerebrovascular disease (including stroke) and ischemic heart disease are the leading causes of deaths in Vietnam [6]. Indeed, the number of people suffering from CVDs has been increasing yearly, with CVDs accounting for about 33% of the total deaths among the Vietnamese population [7,8]. However, while CVDs are highly preventable and treatable, particularly when detected early, accessibility to electrocardiography (ECG) services—the common and widely used method to assess cardiovascular problems—is not available at primary care level in Vietnam and its related cost is high. [4,6]. As a result, it is becoming increasingly important to have easily accessible and affordable procedures at primary care level for early detection of cardiovascular issues to ensure timely intervention and treatment.

There is ongoing biomedical research into the development of portable ECG devices for real-time ECG signal analysis [7-9]. Additionally, feasibility studies have been conducted on a variety of mobile ECG devices [10-14]. Handheld or finger ECG mobile devices currently dominate the portable ECG device market and are widely available for both patient and physician use [10]. Table 1 lists some of the current commercially available handheld mobile ECG devices. Although there are several mobile ECG devices commercially available, they are still inaccessible and unaffordable to most of the general Vietnamese population. Consequently, a mobile ECG device that allows patients to easily navigate their ECG recordings while still being affordable and convenient for use in a Vietnamese context is needed. The MD-Link will be a much more affordable ECG device because it is estimated to be within the price range of US $20-$25 or approximately one-fourth of the current lowest price of handheld ECG devices in the United States. In addition, mobile phone use is ubiquitous in Vietnam, and mobile health (mHealth) implementation is emerging in the country [15], which will thus increase the affordability and practicality of the use of the MD-Link device as it requires a mobile device [16].

### Objective

The main aim of this study is to examine the accuracy of the MD-Link device for detecting irregular heartbeat in a hospital population by comparing some of the standard printed outputs from a 12-lead electrocardiogram generated by the Nihon Kohden Cardiofax S Electrocardiograph Model ECG-1250K, to the calculated standard outputs from the MD-Link 1-lead mobile ECG device [17].

**Table 1:** Commercially available handheld or finger mobile electrocardiography devices.

Device	Price (US $)^a^
AfibAlert, Lohman Technologies, United States	249
ECG Check, CardiacDesigns, United States	139
HeartCheck ECG Pen, CardioComm Solutions, Inc, United States	259
InstantCheck, DailyCare BioMedical Inc, Taiwan	299
Kardia Mobile, AliveCor, Inc, United States	99
ME 90, Beurer GmbH, Germany	120
REKA E100, REKA Health Pte Ltd, Singapore	Not available
WIWE, Sanatmetal, Hungary	438
Zenicor-ECG, Zenicor Medical Systems AB, Sweden	1460

^a^Prices are as indicated on the companies’ websites or as indicated by official distributors.

## Methods

**Figure 1:**
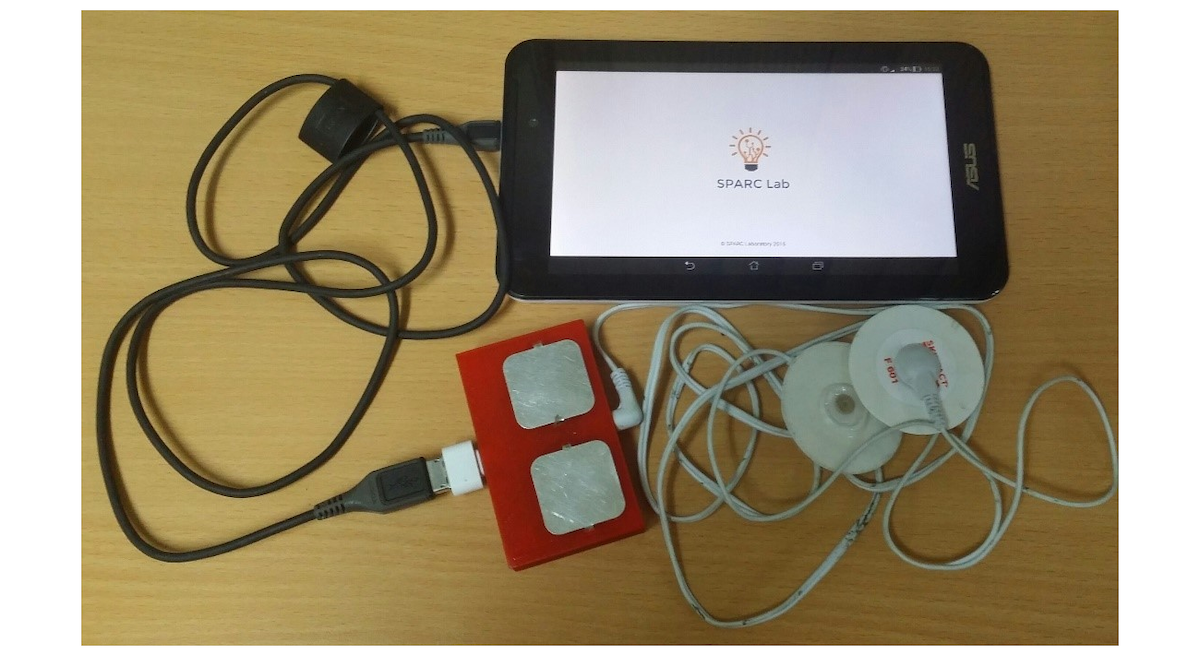
Complete system of the MD-Link mobile electrocardiography device.

The MD-Link system consists of the following 3 key elements: (1) a mobile device (eg, a smartphone or a tablet); (2) a mobile ECG device that can capture ECG signals through either the 2 built-in dry electrodes or a detachable wire system with 2 disposable Ag-AgCl electrodes; and (3) an easy-to-use mobile app interface to display and upload ECG recordings (see Figure 1). As described in [18], the mobile ECG device was designed to measure 1-lead ECG signals; however, it can be easily expanded to measure up to 3 leads—DI, DII, and DIII.

**Figure 2:**
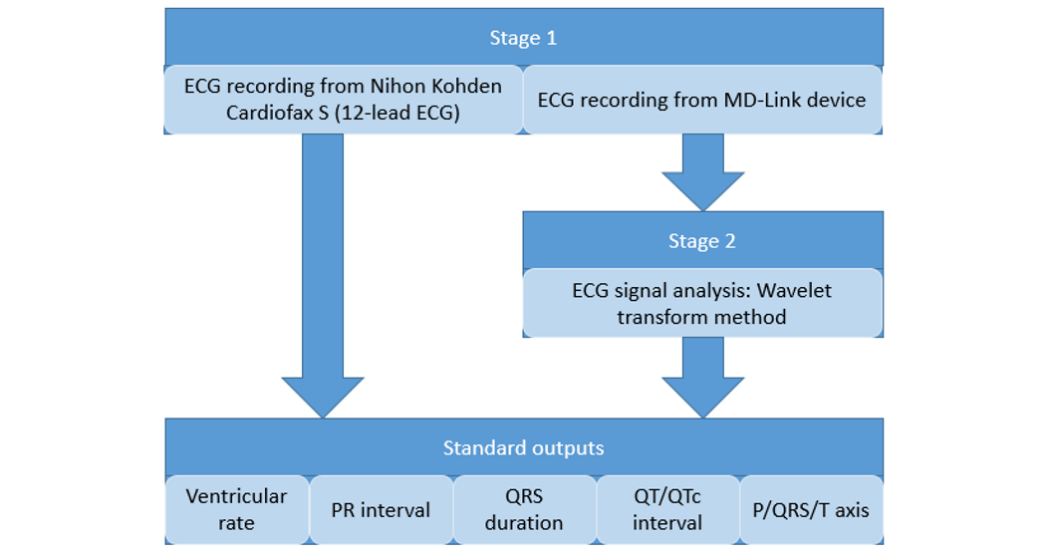
Stages of testing the MD-Link mobile electrocardiography (ECG) device. QTc: corrected QT.

We will conduct this study in two stages. In the first stage, we will measure the ECG signals of the participants using both the MD-Link and the Nihon Kohden Cardiofax S. The MD-Link system will be used first to record 3 leads as follows: (1) DI, which is a signal between the right arm and left arm; (2) DII, which is a signal between the right arm and left leg; and (3) DIII, which is a signal between the left arm and left leg. The Nihon Kohden Cardiofax S will then be used to record a 12-lead ECG. In the second stage, the ECG Viewer software will be used to extract data of the 3 leads (DI, DII, and DIII) from the 12-lead recordings measured by the Nihon Kohden Cardiofax S for comparison against the 3-lead signals captured by the MD-Link system. The wavelet transform method [19-21] will be utilized to calculate clinical standard outputs from the MD-Link system, such as PR interval, QRS duration, QT or corrected QT (QTc) interval, etc, in comparison against the ones provided by the Nihon Kohden Cardiofax S. The final step of analysis will involve the interpretation by two cardiologists to compare the signals and standard outputs of the two systems (see Figure 2).

### Stage 1: Electrocardiographic Measurements

#### Overview

The first step to compare ECG recordings of the MD-Link to the Nihon Kohden Cardiofax S is to take ECG measurements from both devices. We will conduct several on-site visits and will begin our study from July 2018 to October 2018 at Thanh Chan Clinic, a primary care clinic in Hanoi, Vietnam, where ECG measurements will take place.

#### Participants

Subjects will be recruited from among patients with cardiac arrhythmia who receive treatment at Thanh Chan Clinic. To be enrolled in the study, patients must be aged 18 years or older and agree to follow study procedures and provide informed consent. Exclusion criteria include any patient who has a mental health condition sufficiently serious to prevent them from understanding informed assent, including the voluntary nature of participation.

#### Sample Size Determination

As this is a preliminary comparison method study, no formal sample size analysis has been conducted. We anticipate recruiting 30 patients from Thanh Chan Clinic. This number was estimated based on the average number of patients receiving treatment for cardiovascular complications at the clinic.

#### Data Collection

The data acquisition process will proceed in the following 3 steps: (1) an interview before ECG measurement to collect patient demographics and CVD risk factors will be conducted; (2) ECG measurement using the Nihon Kohden Cardiofax S, specifically a full 12-lead ECG recording, will be performed by a trained technician and saved on an SD-card; and (3) successive lead I, lead II, and lead III ECG measurements using the MD-Link mobile device will be performed immediately after step 2. The MD-Link will record the ECG signals for 1 minute per lead and then store data in the app and transfer it to a Web-based software platform by 3G or Wi-Fi. We anticipate the total time for conducting an interview and taking ECG measurements for each patient to be 30 minutes.

#### Ethics

This study has received Institutional Review Board approval from the Committee for the Protection of Human Subjects at Dartmouth College (STUDY00030415) and the Institute of Population, Health and Development in Hanoi, Vietnam (2017/PHAD/ECG-01).

### Stage 2: Data Analysis

#### Overview

The second stage will involve an assessment by two cardiologists and the calculation of the selected clinical standard outputs from the MD-Link in comparison against the ones from the Nihon Kohden Cardiofax S.

We will use the ECG Viewer software to extract DI, DII, and DIII signals from the collected 12-lead ECG signals by the Nihon Kohden Cardiofax S. The wavelet transform method [19-21] will be utilized to calculate standard clinical outputs from the MD-Link system such as PR interval, QRS duration, QT, or QTc interval, etc. The aim of this work is to have ECG signals recorded by two devices with the same indicators. The next step is to export the ECG waves with clinical standard outputs and send to the cardiologists, who are blinded to the devices and results, for clinical interpretation.

Following this, we will compare the final standard outputs from both devices beginning with univariate statistics (mean, median, SD, and 95% CIs) and distribution. We will also analyze the general information and risk factor data collected from the interviews.

#### Analysis

Based on the ECG recordings and outputs of each device, the cardiologists will interpret the results as having an irregular heartbeat or not. Therefore, the sensitivity and specificity of MD-Link and Nihon Kohden Cardiofax S will be calculated and compared. The kappa coefficient will be determined to assess the agreement results between the Nihon Kohden Cardiofax S, the MD-Link, and the cardiologists’ interpretation. A kappa value of more than 0.8 will be considered an “excellent agreement” [22].

Data entry for general information and risk factor data as well as the clinical standard outputs (heart rate, PR interval, etc) will be conducted using the EpiData software (EpiData Association). Stata 12.0 (StataCorp) will be used to perform data analyses. Descriptive statistics will be calculated for patient sociodemographic variables and study outcomes. We plan to use 2-sample *t* tests to compare the clinical standard outputs and kappa values of both devices. Generalized logistic mixed regression including age, risk factors, and medical history will also be used in the analysis. The statistical method results will be considered statistically significant when *P*<.05.

## Results

At the completion of this research, we will have evaluated the accuracy of the MD-Link device in recording ECG signals through the two cardiologists’ interpretations and the comparison of its calculated standard outputs to the printed-out standard outputs of a 12-lead electrocardiogram generated by the Nihon Kohden Cardiofax S. Following this, we will proceed with a publication plan that includes a project report and, ultimately, some articles for peer-reviewed journals. We also hope to disseminate our work at relevant national and international conferences. Recruitment and data collection were completed in January 2018. Data analysis started in February 2018 and is ongoing. Results are expected mid-2019.

## Discussion

### Summary

We propose to conduct a study to investigate the specificity and sensitivity of the MD-Link mobile ECG system in recording ECG measurements, in which the standard outputs are compared against the ones on the clinical-grade Nihon Kohden Cardiofax S system. We expect that the MD-Link will measure and provide 3 ECG lead measurements (DI, DII, and DIII) that are similar to the Nihon Kohden Cardiofax S. The study will also offer participating patients access to their own heartbeat and ECG recordings through a mobile device such as a smartphone or a tablet. The study aims to provide patients with irregular heartbeats a portable solution to monitor changes in their heartbeat over time. Furthermore, patients’ ECG recordings will be synchronized to the cardiologists’ mobile app, which will allow for more timely comprehensive information and interpretation needed for follow-up screenings and diagnoses of the patients.

### Strengths

We have a diverse and multidisciplinary research team whose members are well positioned to successfully complete the proposed study. We have extensive experience in successfully developing and piloting several mHealth projects in recent years as well as implementing these projects in the general Vietnamese population. We have a track record of engaging both local and foreign partners to the success of our mHealth interventions. Importantly, our patient partners and local stakeholders have been engaged from the beginning of the project, and they represent a wide range of perspectives, including our target populations; clinician partners; those with knowledge of mobile technology, hardware engineering, and software development; and those experienced with carrying out public health interventions.

### Limitations

There are several limitations to the study. First, due to the relatively small number of participants (about 30 patients) we will recruit for the study, possible statistical tests to learn about the significant differences between the two systems are quite limited. Second, there are several challenges related to the ECG data processing techniques of the two systems. ECG measurement results from the Nihon Kohden Cardiofax S, including standard clinical outputs such as PR interval, QRS duration, and P-QRS-T axis, etc, are processed internally and can be read by the ECG Viewer software. However, at this stage, the MD-Link system does not contain such features; therefore, all necessary calculations will need to be conducted externally using some software tools such as MATLAB (MathWorks) or R. As a result, artificial variations might be introduced to the ECG measurement results of the two devices, which would create more problems for the comparison work. Finally, the ability to study only 3 leads (DI, DII, and DIII) instead of all available 12 leads also limits the complete interpretation of the MD-Link system.

### Conclusions

Upon completion of this project, we will have developed and tested the MD-Link, a low-cost mobile ECG monitoring device, which will be ready for large-scale testing. Our long-term goal is to make the MD-Link system easily accessible and affordable to the Vietnamese community. Moreover, we hope the device will be used widely at the primary care level and seamlessly fit into an arrhythmia patient’s daily routine to leverage the treatment for patients with cardiovascular health issues and to increase the transparency of care between doctors and patients.

## Abbreviations

CVD: cardiovascular disease
ECG: electrocardiography
mHealth: mobile health
QTc: corrected QT
